# Transition to practice curriculum for general internal medicine physicians: scoping review and Canadian national survey

**DOI:** 10.1186/s12909-022-03673-4

**Published:** 2022-08-09

**Authors:** Benjamin Thomson, Heather O’Halloran, Luke Wu, Stephen Gauthier, David Taylor

**Affiliations:** 1 Department of Medicine, Division of General Internal Medicine, Queen’s University, 76 Stuart Street, Kingston, ON K7L-2V7 Canada; 2 Johns Hopkins Bloomberg School of Public Health, 615 N Wolfe Street, Baltimore, MD 21205 USA

**Keywords:** Competency-based medical education, Transition to practice, Medical Education, Independent practice

## Abstract

**Background:**

There remains a paucity of evidence for curricula for the transition to practice (TTP) stage of Competence by Design internal medicine (IM) training programs. Current entrustable professional activities are based on expert consensus rather than robust subspecialty-specific needs assessment.

**Methods:**

A scoping review was completed to identify studies with TTP focus. A national survey was conducted to identify transition experiences for general internal medicine physicians. Results were assessed by grounded theory analysis to identify core topics for TTP curricula.

**Results:**

Neither scoping review nor national survey identified TTP topics related to the CanMEDS Medical Expert role. *Scoping Review:* 41 relevant studies were identified. Most (97.6%) were from North America. The most common study types were observational (survey) or curriculum (13/41 31.7% for each). Only two studies were exclusively in IM, and the most common subspecialty studied was surgical (13/41, 31.7%). The most common TTP topics were mentorship, billing and coding, practice management, negotiating contract and job, and financial aspects of practice. *National Survey:* There were 44 respondents, with the majority (25/44, 56.8%) having completed an IM subspecialty fellowship. Most (38/44) completed medical school in Canada, and most were from academic practice settings (33/44, 75.0%). The most common TTP topics were billing and coding, personal financial planning, practice management, work-life balance and mentorship. *Grounded Theory Analysis:* There were six themes that encompassed all TTP topics from the scoping review and national survey, being (i) building a career, (ii) continuing professional development, (iii) expectations of the profession, (iv) practice management, (v) Life, health and well-being and (vi) clinical skills. Curriculum competencies and resources for curriculum development were provided.

**Conclusions:**

This study identifies topics critical for curricula development for IM transition to practice. Further research is required to evaluate effectiveness of curricula including topics and themes developed from this scoping review and national survey.

**Supplementary Information:**

The online version contains supplementary material available at 10.1186/s12909-022-03673-4.

## Background

The Royal College of Physicians and Surgeons of Canada, [1] along with other national medical education authorities, [2, 3] have adopted Competency Based Medical Education (CBME) to reform the training of medical specialists. Within Competence by Design (CBD), the Royal College’s approach to CBME, postgraduate learners progress through graduated stages of training in preparation for entering unsupervised practice [4]. The final stage of training in CBD, Transition to Practice (TTP), focuses on the challenges graduating residents face when entering unsupervised practice. An effective TTP curriculum should prepare residents to negotiate these challenges ably and to transition smoothly into practice. It follows that fundamental to building an effective TTP curriculum is a clear understanding of the needs that physicians have in this transition.

Extensive surgical literature describes challenges faced by surgical trainees entering practice [5–9]. However, there is a paucity of literature for internal medicine physicians, making the required curricular components for this stage uncertain. The entrustable professional activities (EPA) adopted and currently used to direct learning and assessment at this stage of residency training are based on expert consensus of clinicians and educators and may not capture the transition needs of internists entering practice [10]. Ensuring a TTP curriculum serves its purpose requires a more robust and specialty-specific needs assessment on which a curriculum can be built.

In constructing a novel Transition to Practice curriculum for the Queen’s Internal Medicine residency program, we sought a robust understanding of the transition experience from residency to unsupervised practice. To achieve this, a scoping review was performed to identify the core themes and interventions previously published with a focus on those relevant to an internal medicine TTP curriculum. To determine if the findings of the scoping review were consistent with general internists’ transition experiences, a national survey of general internists was conducted. Informed by the scoping review, and validated by the national survey, core TTP topics for a curriculum were identified for residents in internal medicine residency training.

## Methods

### Part 1: scoping review

#### Search strategy and study selection criteria

Authors agreed that a scoping review format was optimal, given the broad and exploratory nature of the research question “what is known from the literature about curricula for transition to practice, for physicians wanting to work as general internal medicine physicians?”.

A scoping review was conducted using Cochrane, Embase, Pubmed/Medline and Web of Science databases, with no date or language restriction. Searches were performed June 28 (2021). Search terms included “transition to practice,” “competency based medical education,” “starting independent practice,” “starting practice.” Editorials, commentaries and review articles were excluded, but were read to identify additional studies to include in the scoping review.

##### Inclusion criteria

Studies were included if the study population included either residents or physicians within the first 5 years of unsupervised practice. Observational and interventional trials were included, when the study focus was on transition from supervised to unsupervised practice. Supervised practice was as part of a residency training program, and unsupervised practice was as a licensed practising physician outside of a residency training program. All physician specialties and countries of study were included.

##### Exclusion criteria

Studies that described the transition to practice for health care professionals other than physicians were excluded.

Two authors (BT, LW) screened all titles and abstracts, and two authors (BT, HO) completed full text review, using Covidence systematic review software (Veritas Health Innovation, Melbourne, Australia). Conflicts were resolved by discussion and consensus between both authors.

#### Data extraction

All studies were described by study type, country, population, and subspecialty. All interventional studies were further described by the type and outcome of intervention. Topics identified as important to TTP for each study were recorded.

Meta-analysis of data was not possible due to the heterogenous nature of available studies.

### Part 2: national survey

#### Survey creation

A 9-question survey was created for physicians in unsupervised practice in Canada in internal medicine (IM) (Appendix 1). The overarching reason for the national survey was to deteremine if the scoping review findings were consistent with TTP experiences of a representative cohort of general internists. Survey construction addressed two objectives. Objective 1 was to gather background demographic and training information of survey respondents, to establish the nature of clinical practice and the breadth of representation across the range of IM practices. Objective 2 was to describe survey respondents’ perceptions of challenges faced moving into unsupervised practice, and to determine what might have improved that experience. The survey included one open-ended question (objective 2), followed by 8 multiple choice questions (objective 1). The survey was reviewed and completed by two physicians not involved in the research study, to establish face validity.

#### Participant identification

We sampled internal medicine program directors across Canada and then used snowball sampling to identify general internists in community practice. We aimed to include participants representing diverse backgrounds, practice types, years of experience and geography.

#### Survey distribution and data collection

The survey was distributed electronically using the online survey tool SurveyMonkey (SurveyMonkey, San Mateo, California, USA). A web link to the survey was emailed to program directors in Canada with a request that the invitation email (and link) be shared with other internal medicine physicians in Canada. The survey opened December 3 (2020) and closed on January 30 (2021). Data was collected anonymously. Survey respondents who did not identify as an internal medicine physician in unsupervised practice were excluded.

### Part 3: grounded theory analysis

The results of the scoping review and national survey were assessed using grounded theory and constant comparative method, [11] as previously described.[12] Scoping review foci were identified, coded and organized into a table. Codes were examined and compared to identify connections to create categories. Categories were compared to validate similarities and relationships, then final categories were developed. Two authors (DT, BT) independently created the codes, then defined and named categories. The two authors (DT, BT) discussed their independently defined and named themes, and created a final list after discussion and consensus.

National survey responses foci were then identified, coded and organized into a table. Codes were examined and compared to identify connections to create categories. The national survey codes were then used for theoretical sampling of the codes and themes generated from the scoping review, using the constant comparative method, as previously validated and described [11].

Curricular competencies were identified (DT, BT), initially independently, then finalized after discussion and consensus, to reflect all coded topics.

#### Context of internal medicine residency training program

Queen’s University (Kingston, Ontario, Canada) is one of 17 Canadian internal medicine (IM) residency training programs. Each IM training program takes approximately 3 years, and is separated into stages of Competence by Design (CBD), as per the Royal College of Physicians and Surgeons of Canada (RCPSC) version of Competency Based Medical Education (CBME). The CBD stages include, in order, transition to discipline, foundations of discipline, core of discipline, then transition to practice (TTP). Graduates of Queen’s University’s IM residency training program move into independent practice in a variety of community and academic settings, in urban and rural settings, predominantly throughout Canada.

#### Ethics

The Queen’s University Research Ethics Board provided an exemption for research ethics review for this project, since it fell within the Tri-Council Policy Statement (TCPS)-2 articles 2.4 and 2.5. PROSPERO does not currently register scoping reviews, and as such this study was not registered. However, study data is available on request to the primary author (BT).

## Results

### Part 1: scoping review

**Fig. 1:**
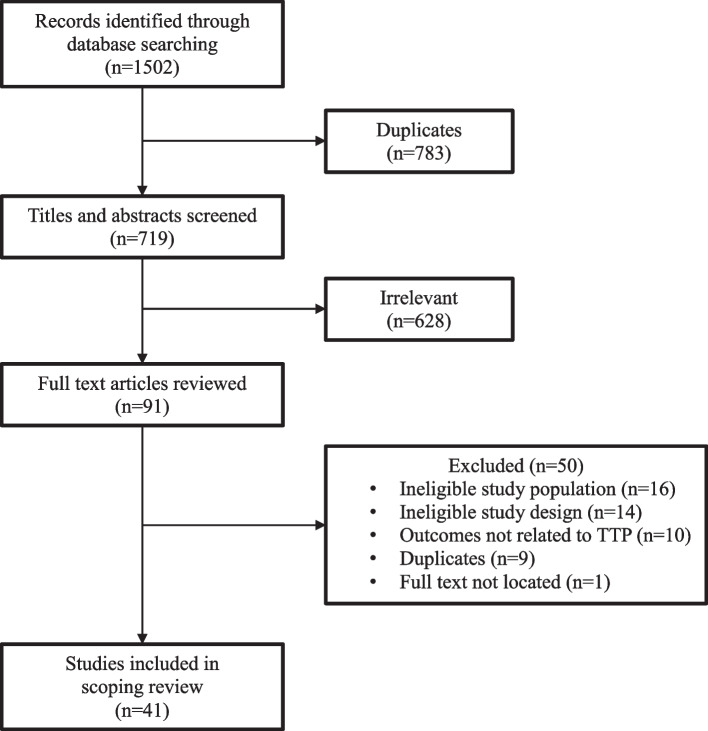
PRISMA Scoping Review Flow Chart

Database search produced 1502 references (Fig. 1). Removal of duplicates (*n* = 783) yielded 719 titles and abstracts to screen. After irrelevant (*n* = 628) titles and abstracts were removed, there were 91 full text articles for review. There were 50 full text articles excluded on full review, leaving 41 studies that were included in this review. The study summary is included (Supplemental Table 1).

**Table 1:** Details of Studies from Scoping Review

Study Characteristic	N (%)
***Country***	
United States	25 (61.0)
Canada	15 (36.6)
Australia	1 (2.4)
***Study Type***	
**Descriptive:** Survey	13 (31.7)
Interview	6 (14.6)
Meetings	1 (2.4)
Review of social media posts	1 (2.4)
Moderated focus groups	1 (2.4)
**Interventional:** Curriculum	13 (31.)
Elective rotation	2 (4.9)
Workshop	1 (2.4)
Journal club with discussion groups	1 (2.4)
Resident run clinic	1 (2.4)
Transition to practice year	1 (2.4)
***Study Population—experience***	
Residents	21 (51.2)
New-to-practice physicians	17 (41.5)
Both residents and new-to-practice physicians	3 (7.3)
***Study Population—subspecialty***	
Surgical subspecialties	13 (31.7)
Pediatrics	6 (14.6)
Emergency medicine	4 (9.8)
Anesthesiology	4 (9.8)
Medicine subspecialties	4 (9.8)
Psychiatry	3 (7.3)
Not specified	3 (7.3)
Internal Medicine	2 (4.9)
Family medicine	1 (2.4)
Radiation oncology	1 (2.4)

#### Study characteristics

Most studies were performed in North America (40/41)(Table 1). Most (22/41) studies were descriptive, with survey being the most common type (*n* = 13) of descriptive study. The remaining studies (19/41) were interventional, and the most common intervention was a curriculum (*n* = 13), followed by elective rotation (*n* = 2).

The study population experience level included residents (*n* = 21), new-to-practice physicians (*n* = 17) or mixed experience level (*n* = 3).

The most common study population subspecialty was surgical (*n* = 13). A small number of studies were from medicine subspecialties (*n* = 4) or general internal medicine (*n* = 3).

#### Outcome characteristics

All included studies identified topics relevant for TTP. Topics relevant to TTP identified from manuscripts in the scoping review Table were summarized (Table 2). The most common topics identified were mentorship (*n* = 12, 29.3%), billing and coding (*n* = 12, 29.3%), practice management (*n* = 11, 26.8%), negotiating for a job or contract (*n* = 10, 24.4%), and financial aspects of practice (*n* = 9, 22.0%). There were 37 topics identified in the scoping review, with frequency ranging from 1 to 13 of the manuscripts.

**Table 2:** Coded Transition to Practice topics from Scoping Review and National Survey

	**Scoping Review**	**National Survey**
**Coded data**	**Count (%)**	**Count (%)**
1.Mentorship	12 (29.3)	7 (15.9)
2. Billing and coding	12 (29.3)	25 (56.8)
3. Practice management	11 (26.8)	11 (25.0)
4. Negotiating contract and job	10 (24.4)	6 (13.6)
5. Personal financial planning	7 (17.1)	18 (40.9)
6. Finding jobs	6 (14.6)	5 (11.4)
7. Work-life balance	5 (12.2)	8 (18.2)
8. Local hospital/health system guidelines/policies	5 (12.2)	5 (11.4)
9. Increased autonomy during residency	4 (9.8)	2 (4.5)
10. Medicolegal issues	4 (9.8)	2 (4.5)
11. Teaching Learners	3 (7.3)	1 (2.3)
12. Documentation	3 (7.3)	1 (2.3)
13. Insurance (personal health/life/disability)	3 (7.3)	1 (2.3)
14. Leadership skills	3 (7.3)	1 (2.3)
15. Outpatient management	1 (2.4)	5 (11.4)
16. Payment/salary systems	1 (2.4)	2 (4.5)
17. Building a CV	2 (4.9)	2 (4.5)
18. Effective communication with colleagues	1 (2.4)	1 (2.3)
19. Continuous Professional Education	1 (2.4)	1 (2.3)
20. Financial aspects of practice	9 (22.0)	0 (0.0)
21. Collaboration with Multidisciplinary team	6 (14.6)	0 (0.0)
22. Interviewing	4 (9.8)	0 (0.0)
23. Making a CV or cover letter	3 (7.3)	0 (0.0)
24. Resources for transition to practice	2 (4.9)	0 (0.0)
25. Quality Assurance	2 (4.9)	0 (0.0)
26. Presenting to public	1 (2.4)	0 (0.0)
27. Advocacy	1 (2.4)	0 (0.0)
28. Outpatient resources	1 (2.4)	0 (0.0)
29. Social media-Professionalism	1 (2.4)	0 (0.0)
30. Handovers	1 (2.4)	0 (0.0)
31. Conflict resolution	1 (2.4)	0 (0.0)
32. Breaking bad news	1 (2.4)	0 (0.0)
33. Reporting obligations	1 (2.4)	0 (0.0)
34. Difficult family management	1 (2.4)	0 (0.0)
35. Informed consent	1 (2.4)	0 (0.0)
36. Choosing between academic versus community	1 (2.4)	0 (0.0)
37. Setting up community services for patients	1 (2.4)	0 (0.0)
38. Difficult Patient conversations	0 (0.0)	1 (2.3)
39. Urban versus rural job settings	0 (0.0)	1 (2.3)
40. Collegiality	0 (0.0)	1 (2.3)

### Part 2: national survey

#### Objective 1: representative sample of general internal medicine physicians

Two sets of survey responses were excluded as the respondent was not practicing as a general internal medicine physician. The remaining survey responses (*n* = 44) were included for further analysis (Table 3).

**Table 3:** National Survey of General Internal Medicine Physicians about Transition to Practice

**Respondent Factor**	**Description**		N (%)
Subspecialty of Practice	General Internal Medicine		44 (100.0)
Educational Background	Subspecialty in GIM	Yes	25(56.8)
		No	19 (43.2)
	Medical School location	Canada	38 (86.4)
		Outside Canada and USA	5 (11.4)
		USA	1 (2.3)
	Core internal medicine residency location	Canada—Ontario	30 (68.2)
		Canada- British Columbia	8 (18.2)
		Canada—Quebec	2 (4.5)
		USA	2 (4.5)
		Canada—Nova Scotia	1 (2.3)
		Canada—Alberta	1 (2.3)
Professional Experience	Age	26–30	2 (4.5)
		31–35	13 (29.5)
		36–40	11 (25.0)
		Greater than 40	18 (40.9)
	Years in independent practice	0–5	17 (38.6)
		6–10	7 (15.9)
		Greater than 10	20 (45.5)
Clinical settings		Academic	33 (75.0)
		Community	10 (22.7)
		Other	1 (2.3)

The survey respondents came from a diverse educational, professional and clinical backgrounds. Most respondents (38/44, 86.4%) completed medical school in Canada. Respondents completed core internal medicine in British Columbia (8/44), Alberta (1/44), Ontario (30/44), Quebec (2/44) and Nova Scotia (1/44). There were approximately equal numbers of internal medicine practitioners who had (25/44) and hadn’t (19/44) completed General Internal Medicine subspecialty training.

There was representation from broad ranges of age, with most respondents between the age of 31–40 (24/44, 54.5%). There was approximately equal representation of internal medicine physicians within the first five years (17/44, 38.6%) and more than 10 years in unsupervised practice (20/44, 45.5%). Most respondents (33/44, 75.0%) were from academic centers, and there was good representation from community internal medicine practitioners (10/44, 22.7%).

#### Objective 2: topics important for transition to practice

The national survey identified 22 different topics relevant to TTP (Table 2). The most frequently identified topics were billing and coding (*n* = 25, 56.8%), personal financial planning (*n* = 18, 40.9%), practice management (*n* = 11, 25.0%), work-life balance (n- = 8, 18.2%) and mentorship (*n* = 7, 15.9%). Most (19/22) national Survey TTP topics were also identified in the scoping review. However, the importance of collegiality, deciding between urban and rural job settings, and difficult patient conversations were identified in the national survey but not the scoping review.

### Part 3: grounded theory analysis

After discussion and consensus, analysis of the coded topics (Table 2) yielded themes and subthemes (Table 4).

**Table 4:** Coded Transition to Practice Topics from Scoping Review and National Survey

THEME	SUB-THEME	CODE (Table 2)	CURRICULAR COMPETENCIES
**Building a Career**	Career mentorship	1,17	Develop a career plan for promotions and career building
	Career mentorship	1,8	Learn the policies and protocols for the hospital and health system
	Career mentorship	14	Identify leadership opportunities and develop a personal leadership plan
	Job application skills	23	Create a personal curriculum vitae and cover letter
	Job application skills	22	Identify the interview process and receive interview feedback prior to formal interviews
	Job application skills	6	Find available jobs
	Job application skills	4	Negotiate a job/contract
	Identification of desired job	39	Determine how to choose between urban and rural location job
	Identification of desired job	36	Determine how to choose between academic and community practice job
	Identification of desired job	16	Learn the differences between salary/payment systems
**Continuing Professional Development**	Continuous professional education	19	Identify learning needs in clinical practice and create personal learning plan
	Professional bodies and memberships	24	Resources for licensing, education, and professional associations
**Expectations of Profession**	Medicolegal	10, 12	Learn and to demonstrate best practice for effective documentation
	Medicolegal	10, 33	Learn local reporting obligations
	Medicolegal	35	Demonstrate best practice for informed consent
	Professional conduct	29	Learn and demonstrate best practice for social media use
	Professional conduct	40	Describe the importance of collegiality
	Professional conduct	26	Demonstrate effective methods to presenting to the public
	Professional conduct	27	Describe and demonstrate effective methods of advocacy
	Quality Improvement in practice	25	Identify and analyze system level safety
	Teaching/supervising role	11	Demonstrate how to be an effective teacher, and how to provide effective feedback to learners
**Practice Management**	Practice finances	3, 2	Demonstrate competence at billing and coding
	Practice finances	3, 20	Identify financial aspects of managing a practice
	Strategy for learning in TTP	9,15	Greater autonomy during residency with resident-run clinics
**Life, Health and Well-being**	Money	5,13	Develop a personal financial plan, including understanding of debt management/life,health and disability insurance, and incorporation
	Person	7	Develop a personal and professional priority and action plan, for work-life balance
**Clinical Skills**		30	Learn and demonstrate best practice for patient signovers
		18	Demonstrate effective communication with colleagues
		21,37	Demonstrate understanding of the roles of members of the multidisciplinary team, demonstrate understanding of their role in transitioning patients safely into community care
		31	Identify common techniques for conflict resolution
		32	Demonstrate best practice for breaking bad news to patients
		34	Demonstrate strategies for managing challenging family members
		38	Demonstrate best practice for difficult patient conversations
		28, 21	Demonstrate understanding of available outpatient patient resources

There were six major TTP themes. Building a career included 12 coded topics relevant to mentorship, job application skills, and identification of a desired job. Continuing professional development included 2 coded topics relevant to continuous professional education and understanding professional bodies and membership groups. Expectations of the profession included 10 coded topics and was subcategorized into medicolegal issues, professional conduct, qualify improvement in practice, and the teaching or supervisory role. Practice management included 5 coded topics related to practice finances and a strategy for learning in transition to practice. Life, Health and Well-being included 3 coded topics related to personal finances and health and well-being. Clinical skills included 9 coded topics related to communication to colleagues and patients, conflict resolution, signovers, and understanding the function of members of the multidisciplinary team.

#### Agreement between scoping review and national survey

The majority (19/22, 86.4%) of topics identified in the national survey were also identified in the scoping review. Three topics were unique to the national survey (difficult patient conversations, urban versus rural job settings, collegiality) but each was identified only once. There was significant overlap in the most commonly identified topics in the scoping review and national survey, with mentorship (29.3 vs 15.9%), billing and coding (29.3 vs 56.8%), practice management (26.0 vs 25.0%), negotiating contract and job (24.4 vs 13.6%) and personal financial planning (17.1 vs 40.9%). There were 18 topics unique to the scoping review, but the majority (12/18, 66.7%) were identified in only one study.

## Discussion

There remains a paucity of literature evaluating internal medicine physicians’ transition to practice within the CBD framework. There are several reasons why medical educators may have overlooked the importance of this transition. Physicians value independent decision-making as a critical part of the medical profession, [13] so transition interventions may be perceived as being overly obtrusive or paternalistic. Similarly, many faculty members successfully transitioned into unsupervised practice when support or guidance may not have been offered, and may thus feel that residents could similarly advance with minimal guidance [14]. However, there is good evidence that residents entering unsupervised practice feel unprepared, [15] that their personal relationships and quality of life suffer, and that this lack of preparation leads some physicians to leave the profession [16–18]. Thus, it remains critical to improve residents’ transition to unsupervised practice.

No manuscript identified in the scoping review, and no response to the national survey suggested that additional time should be spent furthering medical knowledge during the TTP stage of residency training. Using the CanMEDS framework, [19] the Medical Expert role appears to be well taught in internal medicine programs and assessed on certification examinations. Residents nearing the end of training in Canada and the United States consistently feel confident in their clinical knowledge [20–24]. While this may reflect the Dunning-Kruger effect, [25] the earlier CBD stages of Foundations of Training and Core of Discipline largely focus on building their clinical medical expertise. In contrast, the scoping review and National survey identified other CanMEDS roles as needing increased focus for Transition to Practice.

Personal financial planning was frequently identified in the scoping review (17.1%) and national survey (40.9%). Residents commonly are burdened by high levels of debt, which can influence decisions about career direction [26]. Debt associates with poor quality of life, burnout, and medical errors, while also delaying decisions on having children or purchasing a house [27, 28]. Furthermore, there remains minimal study in how to overcome racial and gender disparities in physician career progression and pay [29, 30]. It is thus understandable that this topic is of such importance to physicians when entering unsupervised practice. A Practice Management Seminar was evaluated in Psychiatry residents that involved talks from a financial analyst regarding concepts of return on investment and reimbursement policies. This seminar series was highly rated by participants and may be one mechanism to teach this important TTP topic. Based on the national surveys, personal financial planning education should also cover debt management, life and disability insurance, and the benefits of incorporation. Meeting individually with a financial analyst may have the benefit of personalizing the information with development of a long-term personal financial plan.

Understanding the financial aspects of practice was commonly identified in the scoping review. There are several components to this, including billing and coding, understanding payment or salary systems, as well as the financial components of office management. Formal business training is not commonly incorporated into residency training, leaving most young physicians feeling unprepared to manage the business aspects of practice [31]. The lack of formal business acumen has been described in internal medicine, psychiatry, surgery and anesthesiology, [32–35] and despite wide spread recognition of its importance, program directors agree that trainees remain insufficiently trained in the topic [36]. Business of medicine courses have been widely implemented into obstetrics and gynecology, [37] ophthalmology, [38] surgery, [39] psychiatry, [40] pediatrics, [41] radiology, [42] and internal medicine [43, 44]. Active learning methodologies appear more effective than didactic teaching in family medicine trainees [45]. Further study is thus required to identify the optimal mechanism of delivering this knowledge for internal medicine physicians.

Mentorship is associated with improved patient care and safety, improved physician confidence, job satisfaction, and decreased burnout [46–49]. However, mentorship was the most commonly identified TTP topic in our scoping review (29.3%) and was also commonly identified from the national survey (15.9%). It is likely that a long-term mentor could address many topics identified in this study, including finding jobs, medicolegal issues, leadership skills, building a curriculum vitae, improving communication with colleagues, finding resources for transition to practice, the importance of collegiality, and improving understanding of hospital or health care system guidelines or policies. Unfortunately, the lack of formal mentorship remains a limitation in many training programs. There are many reported reasons for this, including limited time, [50] perceived competition from or financial costs to mentors, [51] and the inability to match race and gender of mentor and mentee [52]. Given the certainty of the mentee benefits, further study is essential to identify how best to incorporate mentorship programs within internal medicine TTP curricula.

The process to become a board certified general internal medicine physician differs between the United States and Canada. In the United States, physicians become board certified, and work unsupervised in internal medicine, after a three-year residency training program. In Canada, after a similar three-year residency training program, physicians must complete a minimum of 1 year of additional training to work unsupervised in an internal medicine setting, and a minimum of 2 years of additional training to achieve a higher level of board certification for general internal medicine subspecialty. These differences in training affect the timing of delivery of the CBME stages of transition to discipline, foundations of discipline and core of discipline, for general internal medicine physicians. There are also likely differences in the clinical practice of general internal medicine physicians in Canada and the United States. However, the focus of this study was the transition to practice stage, the very last stage before starting unsupervised practice. The scoping review’s studies were mostly from Canada (*n* = 15, 37%) and United States (*n* = 25, 61%), with similar results from both Countries. Therefore, despite the different training systems, this study’s conclusions represent research findings from both Canada, and the United States.

Grounded theory analysis provides direction towards the development and evaluation of internal medicine TTP curricula. Three manuscripts in the scoping review described interventions that included internal medicine residents or physicians. In *MacMillan *et al. [53] general internal medicine fellowship graduates were part of a journal club for the purpose of peer mentorship. At each journal club session, a TTP topic was raised such as billing, job negotiations and developing a CV. Relevant TTP topics were identified by an analysis of the sessions. Unfortunately, there was no evaluation of the sessions by attendees, nor was it compared to an alternative method of delivery TTP curricula. In *Kleinschmidt *et al. [54] a TTP curriculum was provided during a 2 week clinical service to internal medicine residents in a USA academic center. Curriculum topics were determined by a survey of an unreported number of recent graduates of the same internal medicine program. Resident participants reported improved abilities in 5 of the 6 areas of focus of the curriculum (leading a team, in-basket management, chronic disease management strategies, providing efficient acute care, and billing/coding). Unfortunately, this study was published as a pilot trial of only four residents in a single center, and thus may not be generalizable. In *Gephart *et al. [55] a trial practice management curriculum was provided in a one-day series of workshops, to resident trainees, focusing on debt repayment, billing compliance, medical malpractice, contract negotiations, lifestyle and financial management. A pre- and post-attendance survey confirmed improved confidence in one topic area (contract negotiations). Unfortunately, the rationale for the chosen topic focus was not stated, and trainees from 20 different specialties were included. Given the typical training duration for internal medicine is shorter, and the breadth of clinical practice broader than for other subspecialties, these findings may not be generalizable to internal medicine residents. On the other hand, the scoping review and national survey identified themes from a variety of subspecialties and thus likely better identify TTP themes of a subspecialty with general internal medicine’s breadth, than if scoping review studies were limited to internal medicine alone. That said, there remains a paucity of research on curriculum interventions for TTP for internal medicine training programs.

This study’s combination of scoping review and national survey yields the most comprehensive list of internal medicine TTP topics for curriculum design. Covering the spectrum of the CanMEDS roles that surround Medical Expert, the analysis is a framework to design a curriculum that confronts each of the challenges internal medicine physicians face when entering unsupervised practice. Resources are freely available for several of the TTP topics identified in our study (Supplementary Table 2). A study is ongoing at our center to develop and to evaluate a curriculum that utilizes many of these resources. It remains essential that such studies are published, regardless of whether positive or negative results are produced.

This study has several notable strengths. Firstly, the combination of a scoping review and national survey led to a comprehensive list of TTP topics for internal medicine physicians. The organization of these topics into themes provides a template for curriculum design and evaluation. Secondly, the scoping review identified high yield TTP topics from areas outside but still relevant to internal medicine, while the inclusion of the majority of these topics was subsequently validated by being identified in a national survey of highly representative internal medicine physicians. Thirdly, the analysis is coupled with curricular competencies, permitting simpler evaluation of a TTP curriculum intervention in a CBME learning environment. There are also weaknesses to this study. Firstly, the majority of the national survey respondents identified as being in academic settings, so the TTP topics may be less generalizable to community settings. A response rate could not be quantitated due to the non-discriminative sampling method. However, demographic data did confirm that the respondents were inclusive of a broad spectrum of practicing general internists. Secondly, it is possible that the TTP experiences of general internists in the early part of their career are more relevant. However, this study did not restrict participation depending on timing of independent practice, as authors preferred to be over-inclusive in topic identification. This strategy was validated by the high level of agreement between the scoping review and national survey identified topics. Thirdly, this study was unable to identify the effect of sex, race and socioeconomic background on the unique TTP challenges faced by internal medicine physicians. While this was not the objective of this study, these factors have a pronounced impact on several TTP topics such as mentorship, negotiation of jobs, and personal financial planning. It is essential that design and evaluation of TTP curricula consider these factors. Fourthly, this study a priori limited study to the transition to independent practice experiences of physician health care professionals only. It is possible that inclusion of nursing or other non-physician health care professionals would have increased the breadth of the study findings. Furthermore, the national survey was only performed in Canada, and did not include respondents from settings in the United States, where CBME learning environments are also common. However, it is unlikely that inclusion would have changed the study’s outcomes, since the scoping review did not limit study inclusion by country, and was in high level of agreement to the Canadian national survey. Finally, this study did not report a TTP curriculum, but rather the important topics to be included. While a curricula has been developed, it is not yet evaluated and thus it would be premature to report. However, the value of this study is reporting the topics that should be included in a TTP curriculum; it is the authors’ hope that this work precipitates multiple curricula that will be evaluated and reported.

## Conclusions

This study identifies topics critical for development of a curriculum for internal medicine transition to practice. Further research is required to evaluate the effectiveness of curricula that include topics and themes developed from this scoping review and national survey.

## Supplementary Information

**Additional file 1. **

**Additional file 2. **

**Additional file 3. **

## Data Availability

All data is available on request of the corresponding author.

## Abbreviations

CBD: Competency by Design
CBME: Competency Based Medical Education
CMPA: Canada Medical Protective Association
EPA: Entrustable Professional Activities
IM: Internal Medicine
TTP: Transition to Practice
USA: United States of America
