# Correction to: Transition to practice curriculum for general internal medicine physicians: scoping review and canadian national survey

**DOI:** 10.1186/s12909-023-04317-x

**Published:** 2023-05-14

**Authors:** Benjamin Thomson, Heather O‘Halloran, Luke Wu, Stephen Gauthier, David Taylor

**Affiliations:** 1 Department of Medicine, Division of General Internal Medicine, Queen’s University, 76 Stuart Street, Kingston, ON K7L-2V7 Canada; 2 Johns Hopkins Bloomberg School of Public Health, Queen’s University, 615 N Wolfe Street, Baltimore, MD 21,205 USA

**Correction: BMC Medical Education (2022) 22:609**

10.1186/s12909-022-03673-4.

Following publication of the original article [1], the authors updated the Additional file 2.

The original article has been corrected.
