# Supplementary material for: Transition to practice curriculum for general internal medicine physicians: scoping review and Canadian national survey
Source: BMC Med Educ. 2022 Aug 9;22:609. doi: 10.1186/s12909-022-03673-4 (PMC9361703; doi:10.1186/s12909-022-03673-4)
Supplement: Supplementary file 1 — Additional file 1. [file 12909_2022_3673_MOESM1_ESM.docx]

**APPENDIX 1:**

**Transition to Practice Survey**

1. Consider the time when you transitioned from being a resident trainee, to starting independent practice. Consider all the challenges you had during this transition.

What would you have liked to know back then that would have made this transition easier? (Open ended – please write below)

1. In what subspecialty do you currently practice? (check one)

General Internal Medicine ❑ Other ❑

1. Did you complete subspecialty training after your internal medicine residency? (check one)

Yes ❑ No ❑

1. How many years have you been in independent medical practice? (check one)

0 to 5 ❑

6 to 10 ❑

greater than 10 ❑

1. The setting of my current primary medical practice is (check one):

Academic ❑ Community ❑ Other ❑

1. Where did you complete medical school? (check one)

Canada ❑

United States of America ❑

Outside Canada and USA ❑

1. How old are you? (check one)

20-25 ❑

26-30 ❑

31-35 ❑

36-40 ❑

Greater than 40 years of age ❑

1. Where did you complete your core internal medicine residency? (check one)

Canada – British Columbia ❑

Canada – Alberta ❑

Canada – Manitoba ❑

Canada – Ontario ❑

Canada – Quebec ❑

Canada – Nova Scotia ❑

Canada – Newfoundland ❑

United States of America ❑

Outside Canada and USA ❑

1. Where did you complete your subspecialty training? (check one)

Canada – British Columbia ❑

Canada – Alberta ❑

Canada – Manitoba ❑

Canada – Ontario ❑

Canada – Quebec ❑

Canada – Nova Scotia ❑

Canada – Newfoundland ❑

United States of America ❑

Outside Canada and USA ❑
