# Supplementary material for: Transition to practice curriculum for general internal medicine physicians: scoping review and Canadian national survey
Source: BMC Med Educ. 2022 Aug 9;22:609. doi: 10.1186/s12909-022-03673-4 (PMC9361703; doi:10.1186/s12909-022-03673-4)
Supplement: Supplementary file 2 — Additional file 2. [file 12909_2022_3673_MOESM2_ESM.docx]

| **Study** | **Study Design** | **Country** | **Population Studied** | **Transition to Practice Topics** |
| --- | --- | --- | --- | --- |
| (56) | Descriptive (survey) | USA | Senior (6-15 years, n=161) and new-to-practice (n-111) abdominal transplant surgeons | (i) Mentorship (ii) Increased autonomy during residency |
| (57) | Interventional (curriculum) | USA | Emergency medicine residents (n=7) | (i) Teaching learners, (ii) Increased autonomy during residency |
| (15) | Descriptive (interview) | Canada | Radiation oncology residents (n=9), and new-to-practice radiation oncologists (n=5) | (i) Practice management (ii) Personal financial planning, (iii) Effective communication with colleagues (iv) Collaboration with multidisciplinary team (v) Documentation, (vi) Mentorship, (vii) Resources for transition to practice |
| (14) | Descriptive (interview) | USA | New-to-practice physicians (subspecialty not specified) (n=68) | (i) Work-life balance (ii) Finding jobs (iii) Interviewing (iv) Negotiating contract/job (v) Practice management |
| (58) | Interventional (curriculum) | USA | Emergency medicine residents (n=8) | (i) Making a CV or Cover letter (ii) Interviewing (iii) Negotiating contract/job (iv) Work-life balance (v) Medicolegal issues (vi) Personal financial planning (vii) Billing and coding |
| (59) | Descriptive (interview) | Canada | New-to-practice paediatricians (n=9) | (i) Setting up community services for patients (ii) Practice management (iii) Continuous Professional education (iv) Building a CV |
| (60) | Descriptive (survey) | USA | New-to-practice thoracic surgeons (n=55) | (i) Negotiating contract and job |
| (61, 62) | Interventional (curriculum) | USA | Anesthesiology residents (n=8) | (i) Collaboration with multidisciplinary team (ii) Billing and coding (iii) Insurance (personal/health/life/disability) (iv) Negotiating contract and job (v) Personal financial planning (vi) Work-life balance |
| (63) | Descriptive (survey) | Canada | New-to-practice Allergy and Immunology physicians (number respondents not stated) | (i) Practice management, (ii) Mentorship |
| (64) | Interventional (transition to practice year) | USA | New-to-practice Orthopedic surgeons (n=69) | (i) Practice management (ii) Billing and coding (iii) Documentation |
| (65) | Descriptive (interview) | Canada | New-to-practice surgeons (n=13) | (i) Mentorship |
| (17) | Descriptive (interview) | USA | New-to-practice colorectal surgeons (n=21) | (i) Mentorship |
| (55) | Interventional (curriculum) | USA | General internal medicine residents (n=25) | (i) Negotiating contract and job (ii) Practice management (iii) Personal financial planning |
| (66) | Descriptive (meetings) | Australia | International medical graduate new-to-practice physicians (subspecialty not specified) (n=55) | (i) Medicolegal issues (ii) Insurance (personal health/life/disability) (iii) Difficult family management (iv) Collaboration with Multidisciplinary team (v) Informed consent (v) Local hospital/health system guidelines/policies |
| (32) | Interventional (curriculum) | USA | Anesthesiology residents (n=37 pre- and 15 post-survey) | (i) Finding jobs (ii) Making a CV or cover letter (iii) Interviewing (iv) Billing and coding (v) Local hospital/health system guidelines/policies (vi) Medicolegal issues (vii) Insurance (personal health/life/disability) (viii) Negotiating contract and job (ix) Personal financial planning |
| (67) | Descriptive (moderated focus groups) | Canada | General surgery residents (n=49) | (i) Increased autonomy during residency |
| (68) | Interventional (elective rotation) | USA | Pediatrics residents (n=24) | (i) Billing and coding |
| (69) | Descriptive (interview) | Canada | Emergency medicine residents (n=18) | (i) Teaching learners |
| (54) | Interventional (curriculum) | USA | New-to-practice General Internal Medicine physicians (n=4) | (i) Billing and coding (ii) Documentation (iii) Leadership skills (iv) Outpatient management |
| (70) | Descriptive (review of social media posts) | USA | New-to-practice urologists (n=281) | (i) Social Media - professionalism |
| (71) | Descriptive (survey) | USA | Practicing US Anesthesiologists (n=1199 total, 10 with 0-1 and 407 with 1-10 years independent practice) | (i) Mentorship (ii) Resources for transition to practice |
| (72) | Interventional (curriculum) | USA | Neurosurgery chief residents (n=26) | (i) Billing and coding (ii) Mentorship (iii)Financial aspects of practice (iv) Collaboration with multidisciplinary team |
| (53) | Interventional (Journal club with discussion groups) | Canada | Internal Medicine Fellows and new-to-practice physicians (n=5 to 10 per journal club) | (i) Mentorship |
| (26) | Descriptive (survey) | USA | Neurology residents (n=143) | (i) Billing and Coding (ii) Practice management (iii) Financial aspects of practice |
| (73) | Interventional (curriculum) | USA | Nephrology residents (n=31) | (i) Finding jobs (ii) Personal financial planning (iii) Leadership skills (iv) Practice management |
| (74) | Interventional (curriculum) | USA | New-to-practice neurosurgeons(n=8) | (i) Financial aspects of practice (ii) Billing and coding (iii) Mentorship |
| (75) | Interventional (curriculum) | Canada | Emergency Medicine residents (n=17) | (i) Billing and coding (ii) Quality assurance (iii) Teaching learners (iv) Handovers (v) Conflict resolution (vi) Breaking bad news (vii) Reporting obligations |
| (76) | Descriptive (survey) | USA | Plastic Surgery Residents (PGY2 or above) (n=166) | (i) Financial aspects of practice |
| (77) | Interventional (curriculum) | USA | Urology, Pediatrics and Ob/Gyn residents (n=44 survey responses) | (i) Financial aspects of practice |
| (78) | Descriptive (survey) | Canada | New-to-practice pediatricians (n=200) | (i) Billing and coding (ii) Financial aspects of practice (iii) Work-life balance (iv) Local hospital/health system guidelines/policies |
| (79) | Descriptive (survey) | Canada | New-to-practice pediatricians (n=172) | (i) Mentorship |
| (80) | Interventional (curriculum) | Canada | Family medicine residents (n=27) | (i) Payment/Salary systems (ii) Practice management (iii) Quality assurance (iv) Local hospital/health system guidelines/policies |
| (81) | Descriptive (survey) | Canada | Psychiatry residents (n=105) | (i) Choosing between academic versus community |
| (82) | Descriptive (survey) | USA | New-to-practice Cardiothoracic surgeons (n=61) | (i) Mentorship (ii) Negotiating contract and job (iii) Finding jobs |
| (83) | Descriptive (survey) | USA | New-to-practice general, colorectal, vascular and cardiothoracic surgeons (n=853) | (i) Financial aspects of practice (ii) Practice management (iii) Local hospital/health system guidelines/policies |
| (84) | Interventional (workshop) | Canada | Psychiatry residents (n=unstated) | (i) Finding jobs (ii) Interviewing (iii) Negotiating contract and job |
| (85) | Interventional (elective rotation) | USA | Anesthesiology resident (n=1) | (i) Collaboration with multidisciplinary team (ii) Leadership skills |
| (86) | Descriptive (survey) | USA | Pediatric surgery program directors (n=20) and residents (n=30) | (i) Mentorship (ii) Finding jobs (iii) Negotiating contract and job (iv) Building a CV (v) Making a CV or cover letter (vi) Personal financial planning (vii) Work-life balance |
| (87) | Descriptive (survey) | Canada | Pediatrics residents (n=36) and new-to-practice pediatricians (n=19) | (i) Presenting to the public (ii) Collaboration with the Multidisciplinary team (iii) Financial aspects of practice (iv) Advocacy (v) Outpatient resources |
| (40) | Interventional (curriculum) | USA | Psychiatry residents (n=6) | (i) Financial aspects of practice (ii) Medicolegal issues (iii) Billing and coding (iv) Practice management (v) Negotiating contract and job |
| (88) | Interventional (resident run clinic) | Canada | Urology residents (n=10) | (i) Improved autonomy during residency |

**References**

1. Harris KA, Nousiainen MT, Reznick R. Competency-based resident education-The Canadian perspective. Surgery. 2020;167(4):681-4.

2. Lindeman B, Sarosi GA. Competency-based resident education: The United States perspective. Surgery. 2020;167(5):777-81.

3. Olopade FE, Adaramoye OA, Raji Y, Fasola AO, Olapade-Olaopa EO. Developing a competency-based medical education curriculum for the core basic medical sciences in an African Medical School. Adv Med Educ Pract. 2016;7:389-98.

4. Canada RCoPaSo. Getting started: What is CBD? 2020 [Stages of training: Competence Continuum diagram]. Available from: <https://www.royalcollege.ca/rcsite/documents/cbd/full-meantime-guide-e>.

5. Bell RH, Jr., Biester TW, Tabuenca A, Rhodes RS, Cofer JB, Britt LD, et al. Operative experience of residents in US general surgery programs: a gap between expectation and experience. Ann Surg. 2009;249(5):719-24.

6. Coleman JJ, Esposito TJ, Rozycki GS, Feliciano DV. Early subspecialization and perceived competence in surgical training: are residents ready? J Am Coll Surg. 2013;216(4):764-71; discussion 71-3.

7. George BC, Bohnen JD, Williams RG, Meyerson SL, Schuller MC, Clark MJ, et al. Readiness of US General Surgery Residents for Independent Practice. Ann Surg. 2017;266(4):582-94.

8. Mattar SG, Alseidi AA, Jones DB, Jeyarajah DR, Swanstrom LL, Aye RW, et al. General surgery residency inadequately prepares trainees for fellowship: results of a survey of fellowship program directors. Ann Surg. 2013;258(3):440-9.

9. Teman NR, Gauger PG, Mullan PB, Tarpley JL, Minter RM. Entrustment of general surgery residents in the operating room: factors contributing to provision of resident autonomy. J Am Coll Surg. 2014;219(4):778-87.

10. Taylor DR, Park YS, Smith CA, Karpinski J, Coke W, Tekian A. Creating Entrustable Professional Activities to Assess Internal Medicine Residents in Training: A Mixed-Methods Approach. Ann Intern Med. 2018;168(10):724-9.

11. Kolb SM. Grounded Theory and the Constant Comparative Method: Valid Research Strategies for Educators. Journal of Emerging Trends in Educational Research and Policy Studies. 2012;3(1):83-6.

12. Nowell L, Norris JM, White DE, Moules NJ. Thematic Analysis: Striving to Meet the Trustworthiness Criteria. International Journal of Qualititative Methods. 2017;16(1).

13. Cochrane C. Successful Medical Trainees and Practitioners. In: C.E.Vincent RHCa, editor. Psychosocial Aspects of Medical Training. Springfield, Illinois1971. p. 168-90.

14. Borus JF. The Transition to Practice. Journal of Medical Education. 1982;57(8):593-601.

15. Best LR, Sengupta A, Murphy RJL, de Metz C, Trotter T, Loewen SK, et al. Transition to practice in radiation oncology: Mind the gap. Radiotherapy and Oncology. 2019;138:126-31.

16. Cogbill TH, Shapiro SB. Transition from Training to Surgical Practice. Surg Clin North Am. 2016;96(1):25-33.

17. Donahue CA, Kuhnen AH, Kleiman DA, Marcello PW, Schoetz DJ, Jr., Roberts PL, et al. How to Get Ahead: Early-Career Colorectal Surgeons Reflect on Their First Few Years in Practice. J Surg Educ. 2020.

18. Sanaee L, Nayer M, Takahashi SG. Practical solutions for implementation of Transition to Practice curricula in a competency-based medical education model. Can Med Educ J. 2020;11(4):e39-e50.

19. Frank JR, Danoff D. The CanMEDS initiative: implementing an outcomes-based framework of physician competencies. Med Teach. 2007;29(7):642-7.

20. Dijkstra IS, Pols J, Remmelts P, Rietzschel EF, Cohen-Schotanus J, Brand PL. How educational innovations and attention to competencies in postgraduate medical education relate to preparedness for practice: the key role of the learning environment. Perspect Med Educ. 2015;4(6):300-7.

21. McDonnell PJ, Kirwan TJ, Brinton GS, Golnik KC, Melendez RF, Parke DW, 2nd, et al. Perceptions of recent ophthalmology residency graduates regarding preparation for practice. Ophthalmology. 2007;114(2):387-91.

22. Morrow G, Illing J, Redfern N, Burford B, Kergon C. Are specialist registrars fully prepared for the role of consultant? The Clinical Teacher. 2009;6:87-90.

23. brown JM, Ryland I, Shaw NJ, Graham DR. Working as a newly appointed consultant: A study into the transition from specialist registrar. Br J Hosp Med (Lond). 2009;70(7):410-4.

24. Lynch DC, Pugno P, Beebe DK, Cullison SW, Lin JJ. Family practice graduate preparedness in the six ACGME competency areas: prequel. Fam Med. 2003;35(5):324-9.

25. Dunning D, Johnson D, J E. Why people fail to recognize their own incompetence. Curr Dir Psychol Sci. 2003;12:83-7.

26. Mahajan A, Cahill C, Scharf E, Gupta S, Ahrens S, Joe E, et al. Neurology residency training in 2017: A survey of preparation, perspectives, and plans. Neurology. 2019;92(2):76-83.

27. West CP, Shanafelt TD, Kolars JC. Quality of life, burnout, educational debt, and medical knowledge among internal medicine residents. JAMA. 2011;306(9):952-60.

28. Rohlfing J, Navarro R, Maniya OZ, Hughes BD, Rogalsky DK. Medical student debt and major life choices other than specialty. Med Educ Online. 2014;19:25603.

29. Sterbling HM, Molena D, Rao SR, Stein SL, Litle VR. Initial report on young cardiothoracic surgeons' first job: From searching to securing and the gaps in between. J Thorac Cardiovasc Surg. 2019;158(2):632-41 e3.

30. Sanfey H, Crandall M, Shaughnessy E, Stein SL, Cochran A, Parangi S, et al. Strategies for Identifying and Closing the Gender Salary Gap in Surgery. J Am Coll Surg. 2017;225(2):333-8.

31. Cantor JC, Baker LC, Hughes RG. Preparedness for practice. Young physicians' views of their professional education. JAMA. 1993;270(9):1035-40.

32. Holak EJ, Kaslow O, Pagel PS. Facilitating the transition to practice: a weekend retreat curriculum for business-of-medicine education of United States anesthesiology residents. Journal of Anesthesia. 2010;24(5):807-10.

33. Williams LL. Teaching residents practice-management knowledge and skills: an in vivo experience. Acad Psychiatry. 2009;33(2):135-8.

34. Adiga K, Buss M, Beasley BW. Perceived, actual, and desired knowledge regarding Medicare billing and reimbursement. A national needs assessment survey of internal medicine residents. J Gen Intern Med. 2006;21(5):466-70.

35. Stubbe DE. Preparation for practice: child and adolescent psychiatry graduates' assessment of training experiences. J Am Acad Child Adolesc Psychiatry. 2002;41(2):131-9.

36. Lusco VC, Martinez SA, Polk HC, Jr. Program directors in surgery agree that residents should be formally trained in business and practice management. Am J Surg. 2005;189(1):11-3.

37. Williford LE, Ling FW, Summitt RL, Jr., Stovall TG. Practice management in obstetrics and gynecology residency curriculum. Obstet Gynecol. 1999;94(3):476-9.

38. Tsai JC, Lee PP, Chasteen S, Taylor RJ, Brennan MW, Schmidt GE. Resident physician mentoring program in ophthalmology: the Tennessee experience. Arch Ophthalmol. 2006;124(2):264-7.

39. Gill JB, Schutt RC, Jr. Practice management education in orthopaedic surgical residencies. J Bone Joint Surg Am. 2007;89(1):216-9.

40. Wichman CL, Netzel PJ, Menaker R. Preparing psychiatric residents for the "real world": a practice management curriculum. Acad Psychiatry. 2009;33(2):131-4.

41. Babitch LA. Teaching practice management skills to pediatric residents. Clin Pediatr (Phila). 2006;45(9):846-9.

42. Chan S. Management education during radiology residency: development of an educational practice. Acad Radiol. 2004;11(11):1308-17.

43. David RA, Reich LM. The creation and evaluation of a systems-based practice/managed care curriculum in a primary care internal medicine residency program. Mt Sinai J Med. 2005;72(5):296-9.

44. Crites GE, Schuster RJ. A preliminary report of an educational intervention in practice management. BMC Med Educ. 2004;4:15.

45. Bayard M, Peeples CR, Holt J, David DJ. An interactive approach to teaching practice management to family practice residents. Fam Med. 2003;35(9):622-4.

46. Yardley S, Westerman M, Bartlett M, Walton JM, Smith J, Peile E. The do's, don't and don't knows of supporting transition to more independent practice. Perspect Med Educ. 2018;7(1):8-22.

47. Higgins R, Gallen D, Whiteman S. Meeting the non-clinical education and training needs of new consultants. Postgrad Med J. 2005;81(958):519-23.

48. Sachdeva AK, Flynn TC, Brigham TP, Dacey RG, Jr., Napolitano LM, Bass BL, et al. Interventions to address challenges associated with the transition from residency training to independent surgical practice. Surgery. 2014;155(5):867-82.

49. Griffin A, Abouharb T, Etherington C, Bandura T. Transitional to independent practice: a national enquiry into the educational support for newly qualified GPs. Educ Primary Care. 2010;21:299-307.

50. Ecomopoulos K, Sun R, Garvey E, Ba’zzarelli A. Coaching and mentoring modern surgeons. Bull Am Coll Surg. 2014;99:30-5.

51. Straus SE, Chatur F, Taylor M. Issues in the mentor-mentee relationship in academic medicine: A qualitative study. Acad Med. 2009;2009(84):135-9.

52. Fang D, Moy E, Colburn L, Hurley J. Racial and ethnic disparities in faculty promotion in academic medicine. JAMA. 2000;284:1085-92.

53. MacMillan TE, Rawal S, Cram P, Liu J. A journal club for peer mentorship: helping to navigate the transition to independent practice. Perspectives on Medical Education. 2016;5(5):312-5.

54. Kleinschmidt P, Addington-White J, Feldstein DA, Abraham V, Baier L. Increasing Senior Resident Readiness to Practice in Primary Care. Journal of General Internal Medicine. 2018;33:S712-S3.

55. Gephart MH, Schaffer R, Katznelson L, Piro N. Transition-to-Practice Curriculum in Graduate Medical Education. Journal of Neurosurgery. 2017;126(4):A1398-A.

56. Aboulioud M, Hirose R, Nagai S, Gordon C, Farmer D. Surgeon Readiness for Entry Into Practice: A Survey of Abdominal Transplant Surgeons in the United States. Am J Transplant. 2019;19 (suppl 3).

57. Arno KL, Hock S. Senior Resident “Simtending” Curriculum: Novel Simulation-Based Transition to Practice. Acad Emerg Med. 2020;27(S1):S321.

58. Caretta-Weyer H. Transition to Practice: A Novel Life Skills Curriculum for Emergency Medicine Residents. Western Journal of Emergency Medicine. 2019;20(1):100-4.

59. Chan M, van Manen MA. Exploring the transition into practice of general paediatricians from a Canadian residency program. Paediatr Child Health. 2018;23(5):314-8.

60. Chu D, Vaporciyan AA, Iannettoni MD, Ikonomidis JS, Odell DD, Shemin RJ, et al. Are There Gaps in Current Thoracic Surgery Residency Training Programs? Annals of Thoracic Surgery. 2016;101(6):2350-6.

61. Cooper L, Sinclair D, Cobas M, Freytag A, Grossman J. Transition to Practice: A New Paradigm in Anesthesiology Resident Training. Anesthesia and Analgesia. 2010;110(3):S201.

62. Cooper L, Sinclair D, Cobas M, Freytag A, Grossman J, Manning R. Transition-to-practice: New Training Paradigm Improves Resident Performance of Practice Management Skills. Anesthesia and Analgesia. 2010;110(3):S216.

63. Cyr CE, Cyr MM, Quirt J, Connors L. Transition to practice: lessons learned in allergy and immunology training. Allergy Asthma and Clinical Immunology. 2021;17(S1):17.

64. Daniels AH, McDonnell M, Born CT, Hayda RA, Ehrlich MG, P.G. T, et al. Critical Analysis of a Trauma Fellowship-Modeled, Six-Year Orthopedic Surgery Training Program. Journal of Bone and Joint Surgery-American Volume. 2013;95(15):e108(1) - e (8).

65. de Montbrun S, Patel P, Mobilio MH, Moulton CA. Am I Cut Out for This? Transitioning from Surgical Trainee to Attending

Journal of Surgical Education. 2017;75(3):606-12.

66. Harris AO, Delany C. International medical graduates in transition. The Clinical Teacher. 2013;10:328-32.

67. Huynh C, Wong-Chong N, Vourtzoumis P, Lim S, Marini W, Johal G, et al. The future of general surgery training: A Canadian resident nationwide Delphi consensus statement. Surgery. 2019;166(5):726-34.

68. Kelly M, Posa M. Transition to Pediatric Practice: A Residency Elective Experience to Prepare Senior Pediatric Residents for General Pediatric Primary Care. MedEdPORTAL. 2016;12:10506.

69. Kilbertus S, Pardhan K, Zaheer J, Bandiera G. Transition to practice: Evaluating the need for formal training in supervision and assessment among senior emergency medicine residents and new to practice emergency physicians. Canadian Journal of Emergency Medicine. 2019;21(3):418-26.

70. Koo K, Bowman MS, Ficko Z, Gormley EA. Older and wiser? Changes in unprofessional content on urologists' social media after transition from residency to practice. Bju International. 2018;122(2):337-43.

71. Kuza CM, Harbell MW, Malinzak EB, Goff KL, Bicket MC, Ifeanyi-Pillette IC, et al. Transition to Practice in Anesthesiology: Survey Results of Practicing Anesthesiologists on Their Experience. J Educ Perioper Med. 2019;21(2):E619.

72. Lister JR, Friedman WA, Murad GJ, Dow J, Lombard GJ. Evaluation of a Transition to Practice Program for Neurosurgery Residents: Creating a Safe Transition From Resident to Independent Practitioner. Journal of Graduate Medical Education. 2010;September:366-72.

73. Miracle C, Vargas ER, Mullaney S, Nangia S, Makadia PM. Nephrology business leadership university: Filling a gap in fellowship education. Journal of the American Society of Nephrology. 2018;29:S65-S.

74. Murad G LJ, Friedman WA, Lombard G. Enhncing Competence in Graduates Through a Transition to Practice Program in Neurological Surgery: 907. Neurosurgery. 2009.

75. Nath A, Cheung WJ, Leppard J, Perry JJ. A novel transition to practice curriculum for CCFP(EM) programs. Canadian Journal of Emergency Medicine. 2021;23(3):394-7.

76. Ovadia SA, Gishen K, Desai U, Garcia AM, Thaller SR. Education on the Business of Plastic Surgery During Training: A Survey of Plastic Surgery Residents. Aesthetic Plast Surg. 2018;42(3):886-90.

77. Patel R RK, Barone J, Elsamra SE. Business education for residents: Results of a Pilot Business Course at a Urology Residency Program. Urology Practice. 2018;5(2):107-11.

78. Rowan-Legg A. Canadian Early Career Paediatricians on their Transition to Practice Experience. Paediatr Child Health. 2018;23(S1):e57-e8.

79. Schrewe B, Patel R, Rowan-Legg A. Growth curves: The experiences of Canadian paediatricians in their first 5 years of independent practice. Paediatrics & Child Health. 2020;25(4):235-40.

80. Shortt SED, Hodgetts PG. A curriculum for the times: an experiment in teaching health policy to residents in family medicine. Canadian Medical Association Journal. 1997;157:1567-9.

81. Sockalingam S, Wiljer D, Yufe S, Knox MK, Fefergrad M, Silver I, et al. The Relationship Between Academic Motivation and Lifelong Learning During Residency: A Study of Psychiatry Residents. Acad Med. 2016;91(10):1423-30.

82. Sterbling HM, Molena D, Rao SR, Stein SL, Litle VR. Initial report on young cardiothoracic surgeons’ first job: From searching to securing adn the gaps in between. The Journal of Thoracic and Cardiovascular Surgery. 2019;August(632-639).

83. Stolarski A, He K, Sell N, Chugh P, O'Neal P, Smink DS, et al. Mentoring experience of new surgeons during their transition to independent practice: A nationwide survey. Surgery. 2021;169(6):1354-60.

84. Stratton J, Matheson K, Davidson S. Transition to Practice Workshop: A Novel Interactive Intervention to Improve Resident Education. Journal of the American Academy of Child and Adolescent Psychiatry. 2018;57(10):S197-S.

85. Patel Y, Yen C, Tsai M, Kelbert J, Easdowne J, Macario A. A four week or management rotation utilizing a cRNA-based evaluation tool. Anesthesia and Analgesia. 2012;114(5 Supplement 1):S204.

86. Velazco CS, Davila VJ, Alhajjat AM, Ostlie DJ, Garvey EM. Mentorship in pediatric surgery: A need for structure? Journal of Pediatric Surgery. 2021;56(5):892-9.

87. Vetere P, Cooke S. Preparedness to practice paediatric hospital medicine. Paediatrics & Child Health. 2020;25(7):447-54.

88. Witherspoon L, Jalali S, Roberts MT. Resident-run urology clinics: A tool for use in competency-based medical education for teaching and assessing transition-to-practice skills. Cuaj-Canadian Urological Association Journal. 2019;13(9):E279-E84.

**REFERENCES IN FINAL MANUSCRIPT**

## References

1. Harris KA, Nousiainen MT, Reznick R. Competency-based resident education-the Canadian perspective. *Surgery.*2020;**167**(4):681–684. [[PubMed](https://pubmed.ncbi.nlm.nih.gov/31431292)] [[Google Scholar](https://scholar.google.com/scholar_lookup?journal=Surgery&title=Competency-based+resident+education-the+Canadian+perspective&author=KA+Harris&author=MT+Nousiainen&author=R+Reznick&volume=167&issue=4&publication_year=2020&pages=681-684&pmid=31431292&)]

2. Lindeman B, Sarosi GA. Competency-based resident education: the United States perspective. *Surgery.*2020;**167**(5):777–781. [[PubMed](https://pubmed.ncbi.nlm.nih.gov/31383468)] [[Google Scholar](https://scholar.google.com/scholar_lookup?journal=Surgery&title=Competency-based+resident+education:+the+United+States+perspective&author=B+Lindeman&author=GA+Sarosi&volume=167&issue=5&publication_year=2020&pages=777-781&pmid=31383468&)]

3. Olopade FE, Adaramoye OA, Raji Y, Fasola AO, Olapade-Olaopa EO. Developing a competency-based medical education curriculum for the core basic medical sciences in an African medical school. *Adv Med Educ Pract.*2016;**7**:389–398. [[PMC free article](https://www.ncbi.nlm.nih.gov/pmc/articles/PMC4957633/)] [[PubMed](https://pubmed.ncbi.nlm.nih.gov/27486351)] [[Google Scholar](https://scholar.google.com/scholar_lookup?journal=Adv+Med+Educ+Pract&title=Developing+a+competency-based+medical+education+curriculum+for+the+core+basic+medical+sciences+in+an+African+medical+school&author=FE+Olopade&author=OA+Adaramoye&author=Y+Raji&author=AO+Fasola&author=EO+Olapade-Olaopa&volume=7&publication_year=2016&pages=389-398&pmid=27486351&)]

4. Canada RCoPaSo. Getting started: What is CBD? 2020 [Stages of training: Competence Continuum diagram]. Available from: <https://www.royalcollege.ca/rcsite/documents/cbd/full-meantime-guide-e>.

5. Bell RH, Jr, Biester TW, Tabuenca A, Rhodes RS, Cofer JB, Britt LD, et al. Operative experience of residents in US general surgery programs: a gap between expectation and experience. *Ann Surg.*2009;**249**(5):719–724. [[PubMed](https://pubmed.ncbi.nlm.nih.gov/19387334)] [[Google Scholar](https://scholar.google.com/scholar_lookup?journal=Ann+Surg&title=Operative+experience+of+residents+in+US+general+surgery+programs:+a+gap+between+expectation+and+experience&author=RH+Bell&author=TW+Biester&author=A+Tabuenca&author=RS+Rhodes&author=JB+Cofer&volume=249&issue=5&publication_year=2009&pages=719-724&pmid=19387334&)]

6. Coleman JJ, Esposito TJ, Rozycki GS, Feliciano DV. Early subspecialization and perceived competence in surgical training: are residents ready? *J Am Coll Surg.*2013;**216**(4):764–71. [[PubMed](https://pubmed.ncbi.nlm.nih.gov/23521960)] [[Google Scholar](https://scholar.google.com/scholar_lookup?journal=J+Am+Coll+Surg.&title=Early+subspecialization+and+perceived+competence+in+surgical+training:+are+residents+ready?&author=JJ+Coleman&author=TJ+Esposito&author=GS+Rozycki&author=DV+Feliciano&volume=216&issue=4&publication_year=2013&pages=764-71&pmid=23521960&)]

7. George BC, Bohnen JD, Williams RG, Meyerson SL, Schuller MC, Clark MJ, et al. Readiness of US general surgery residents for independent practice. *Ann Surg.*2017;**266**(4):582–594. [[PubMed](https://pubmed.ncbi.nlm.nih.gov/28742711)] [[Google Scholar](https://scholar.google.com/scholar_lookup?journal=Ann+Surg&title=Readiness+of+US+general+surgery+residents+for+independent+practice&author=BC+George&author=JD+Bohnen&author=RG+Williams&author=SL+Meyerson&author=MC+Schuller&volume=266&issue=4&publication_year=2017&pages=582-594&pmid=28742711&)]

8. Mattar SG, Alseidi AA, Jones DB, Jeyarajah DR, Swanstrom LL, Aye RW, et al. General surgery residency inadequately prepares trainees for fellowship: results of a survey of fellowship program directors. *Ann Surg.*2013;**258**(3):440–449. [[PubMed](https://pubmed.ncbi.nlm.nih.gov/24022436)] [[Google Scholar](https://scholar.google.com/scholar_lookup?journal=Ann+Surg&title=General+surgery+residency+inadequately+prepares+trainees+for+fellowship:+results+of+a+survey+of+fellowship+program+directors&author=SG+Mattar&author=AA+Alseidi&author=DB+Jones&author=DR+Jeyarajah&author=LL+Swanstrom&volume=258&issue=3&publication_year=2013&pages=440-449&pmid=24022436&)]

9. Teman NR, Gauger PG, Mullan PB, Tarpley JL, Minter RM. Entrustment of general surgery residents in the operating room: factors contributing to provision of resident autonomy. *J Am Coll Surg.*2014;**219**(4):778–787. [[PubMed](https://pubmed.ncbi.nlm.nih.gov/25158911)] [[Google Scholar](https://scholar.google.com/scholar_lookup?journal=J+Am+Coll+Surg&title=Entrustment+of+general+surgery+residents+in+the+operating+room:+factors+contributing+to+provision+of+resident+autonomy&author=NR+Teman&author=PG+Gauger&author=PB+Mullan&author=JL+Tarpley&author=RM+Minter&volume=219&issue=4&publication_year=2014&pages=778-787&pmid=25158911&)]

10. Taylor DR, Park YS, Smith CA, Karpinski J, Coke W, Tekian A. Creating entrustable professional activities to assess internal medicine residents in training: a mixed-methods approach. *Ann Intern Med.*2018;**168**(10):724–729. [[PubMed](https://pubmed.ncbi.nlm.nih.gov/29710333)] [[Google Scholar](https://scholar.google.com/scholar_lookup?journal=Ann+Intern+Med&title=Creating+entrustable+professional+activities+to+assess+internal+medicine+residents+in+training:+a+mixed-methods+approach&author=DR+Taylor&author=YS+Park&author=CA+Smith&author=J+Karpinski&author=W+Coke&volume=168&issue=10&publication_year=2018&pages=724-729&pmid=29710333&)]

11. Kolb SM. Grounded theory and the constant comparative method: valid research strategies for educators. *Journal of Emerging Trends in Educational Research and Policy Studies.*2012;**3**(1):83–86. [[Google Scholar](https://scholar.google.com/scholar_lookup?journal=Journal+of+Emerging+Trends+in+Educational+Research+and+Policy+Studies&title=Grounded+theory+and+the+constant+comparative+method:+valid+research+strategies+for+educators&author=SM+Kolb&volume=3&issue=1&publication_year=2012&pages=83-86&)]

12. Nowell L, Norris JM, White DE, Moules NJ. Thematic Analysis: Striving to Meet the Trustworthiness Criteria. Int J Qualititative Methods. 2017;16(1):1–16.

13. Cochrane C. Successful Medical Trainees and Practitioners. In: C.E.Vincent RHCa, editor. *Psychosocial Aspects of Medical Training.* Illinois: Springfield; 1971. pp. 168–90. [[Google Scholar](https://scholar.google.com/scholar_lookup?title=Psychosocial+Aspects+of+Medical+Training&author=C+Cochrane&publication_year=1971&)]

14. Borus JF. The transition to practice. *J Med Educ.*1982;**57**(8):593–601. [[PubMed](https://pubmed.ncbi.nlm.nih.gov/7097732)] [[Google Scholar](https://scholar.google.com/scholar_lookup?journal=J+Med+Educ&title=The+transition+to+practice&author=JF+Borus&volume=57&issue=8&publication_year=1982&pages=593-601&pmid=7097732&)]

15. Best LR, Sengupta A, Murphy RJL, de Metz C, Trotter T, Loewen SK, et al. Transition to practice in radiation oncology: mind the gap. *Radiother Oncol.*2019;**138**:126–131. [[PubMed](https://pubmed.ncbi.nlm.nih.gov/31252294)] [[Google Scholar](https://scholar.google.com/scholar_lookup?journal=Radiother+Oncol&title=Transition+to+practice+in+radiation+oncology:+mind+the+gap&author=LR+Best&author=A+Sengupta&author=RJL+Murphy&author=C+de+Metz&author=T+Trotter&volume=138&publication_year=2019&pages=126-131&pmid=31252294&)]

16. Cogbill TH, Shapiro SB. Transition from training to surgical practice. *Surg Clin North Am.*2016;**96**(1):25–33. [[PubMed](https://pubmed.ncbi.nlm.nih.gov/26612017)] [[Google Scholar](https://scholar.google.com/scholar_lookup?journal=Surg+Clin+North+Am&title=Transition+from+training+to+surgical+practice&author=TH+Cogbill&author=SB+Shapiro&volume=96&issue=1&publication_year=2016&pages=25-33&pmid=26612017&)]

17. Donahue CA, Kuhnen AH, Kleiman DA, Marcello PW, Schoetz DJ, Roberts PL, et al. How to Get Ahead: Early-Career Colorectal Surgeons Reflect on Their First Few Years in Practice. J Surg Educ. 2021;78(1):126–33. [[PubMed](https://pubmed.ncbi.nlm.nih.gov/32660856)]

18. Sanaee L, Nayer M, Takahashi SG. Practical solutions for implementation of Transition to Practice curricula in a competency-based medical education model. *Can Med Educ J.*2020;**11**(4):e39–e50. [[PMC free article](https://www.ncbi.nlm.nih.gov/pmc/articles/PMC7417824/)] [[PubMed](https://pubmed.ncbi.nlm.nih.gov/32821301)] [[Google Scholar](https://scholar.google.com/scholar_lookup?journal=Can+Med+Educ+J&title=Practical+solutions+for+implementation+of+Transition+to+Practice+curricula+in+a+competency-based+medical+education+model&author=L+Sanaee&author=M+Nayer&author=SG+Takahashi&volume=11&issue=4&publication_year=2020&pages=e39-e50&pmid=32821301&)]

19. Frank JR, Danoff D. The CanMEDS initiative: implementing an outcomes-based framework of physician competencies. *Med Teach.*2007;**29**(7):642–647. [[PubMed](https://pubmed.ncbi.nlm.nih.gov/18236250)] [[Google Scholar](https://scholar.google.com/scholar_lookup?journal=Med+Teach&title=The+CanMEDS+initiative:+implementing+an+outcomes-based+framework+of+physician+competencies&author=JR+Frank&author=D+Danoff&volume=29&issue=7&publication_year=2007&pages=642-647&pmid=18236250&)]

20. Dijkstra IS, Pols J, Remmelts P, Rietzschel EF, Cohen-Schotanus J, Brand PL. How educational innovations and attention to competencies in postgraduate medical education relate to preparedness for practice: the key role of the learning environment. *Perspect Med Educ.*2015;**4**(6):300–307. [[PMC free article](https://www.ncbi.nlm.nih.gov/pmc/articles/PMC4673059/)] [[PubMed](https://pubmed.ncbi.nlm.nih.gov/26498596)] [[Google Scholar](https://scholar.google.com/scholar_lookup?journal=Perspect+Med+Educ&title=How+educational+innovations+and+attention+to+competencies+in+postgraduate+medical+education+relate+to+preparedness+for+practice:+the+key+role+of+the+learning+environment&author=IS+Dijkstra&author=J+Pols&author=P+Remmelts&author=EF+Rietzschel&author=J+Cohen-Schotanus&volume=4&issue=6&publication_year=2015&pages=300-307&pmid=26498596&)]

21. McDonnell PJ, Kirwan TJ, Brinton GS, Golnik KC, Melendez RF, Parke DW, 2nd, et al. Perceptions of recent ophthalmology residency graduates regarding preparation for practice. *Ophthalmology.*2007;**114**(2):387–391. [[PubMed](https://pubmed.ncbi.nlm.nih.gov/17187862)] [[Google Scholar](https://scholar.google.com/scholar_lookup?journal=Ophthalmology&title=Perceptions+of+recent+ophthalmology+residency+graduates+regarding+preparation+for+practice&author=PJ+McDonnell&author=TJ+Kirwan&author=GS+Brinton&author=KC+Golnik&author=RF+Melendez&volume=114&issue=2&publication_year=2007&pages=387-391&pmid=17187862&)]

22. Morrow G, Illing J, Redfern N, Burford B, Kergon C. Are specialist registrars fully prepared for the role of consultant? *Clin Teach.*2009;**6**:87–90. [[Google Scholar](https://scholar.google.com/scholar_lookup?journal=Clin+Teach&title=Are+specialist+registrars+fully+prepared+for+the+role+of+consultant?&author=G+Morrow&author=J+Illing&author=N+Redfern&author=B+Burford&author=C+Kergon&volume=6&publication_year=2009&pages=87-90&)]

23. Brown JM, Ryland I, Shaw NJ, Graham DR. Working as a newly appointed consultant: a study into the transition from specialist registrar. *Br J Hosp Med (Lond). 2009.*2009;**70**(7):410. [[PubMed](https://pubmed.ncbi.nlm.nih.gov/19584785)] [[Google Scholar](https://scholar.google.com/scholar_lookup?journal=Br+J+Hosp+Med+(Lond).+2009&title=Working+as+a+newly+appointed+consultant:+a+study+into+the+transition+from+specialist+registrar&author=JM+Brown&author=I+Ryland&author=NJ+Shaw&author=DR+Graham&volume=70&issue=7&publication_year=2009&pages=410&)]

24. Lynch DC, Pugno P, Beebe DK, Cullison SW, Lin JJ. Family practice graduate preparedness in the six ACGME competency areas: prequel. *Fam Med.*2003;**35**(5):324–329. [[PubMed](https://pubmed.ncbi.nlm.nih.gov/12772933)] [[Google Scholar](https://scholar.google.com/scholar_lookup?journal=Fam+Med&title=Family+practice+graduate+preparedness+in+the+six+ACGME+competency+areas:+prequel&author=DC+Lynch&author=P+Pugno&author=DK+Beebe&author=SW+Cullison&author=JJ+Lin&volume=35&issue=5&publication_year=2003&pages=324-329&pmid=12772933&)]

25. Dunning D, Johnson D, Ehrlinger J. Why people fail to recognize their own incompetence. *Curr Dir Psychol Sci.*2003;**12**:83–87. [[Google Scholar](https://scholar.google.com/scholar_lookup?journal=Curr+Dir+Psychol+Sci&title=Why+people+fail+to+recognize+their+own+incompetence&author=D+Dunning&author=D+Johnson&author=J+Ehrlinger&volume=12&publication_year=2003&pages=83-87&)]

26. Mahajan A, Cahill C, Scharf E, Gupta S, Ahrens S, Joe E, et al. Neurology residency training in 2017: A survey of preparation, perspectives, and plans. *Neurology.*2019;**92**(2):76–83. [[PubMed](https://pubmed.ncbi.nlm.nih.gov/30518554)] [[Google Scholar](https://scholar.google.com/scholar_lookup?journal=Neurology&title=Neurology+residency+training+in+2017:+A+survey+of+preparation,+perspectives,+and+plans&author=A+Mahajan&author=C+Cahill&author=E+Scharf&author=S+Gupta&author=S+Ahrens&volume=92&issue=2&publication_year=2019&pages=76-83&pmid=30518554&)]

27. West CP, Shanafelt TD, Kolars JC. Quality of life, burnout, educational debt, and medical knowledge among internal medicine residents. *JAMA.*2011;**306**(9):952–960. [[PubMed](https://pubmed.ncbi.nlm.nih.gov/21900135)] [[Google Scholar](https://scholar.google.com/scholar_lookup?journal=JAMA&title=Quality+of+life,+burnout,+educational+debt,+and+medical+knowledge+among+internal+medicine+residents&author=CP+West&author=TD+Shanafelt&author=JC+Kolars&volume=306&issue=9&publication_year=2011&pages=952-960&pmid=21900135&)]

28. Rohlfing J, Navarro R, Maniya OZ, Hughes BD, Rogalsky DK. Medical student debt and major life choices other than specialty. *Med Educ Online.*2014;**19**:25603. [[PMC free article](https://www.ncbi.nlm.nih.gov/pmc/articles/PMC4229497/)] [[PubMed](https://pubmed.ncbi.nlm.nih.gov/25391976)] [[Google Scholar](https://scholar.google.com/scholar_lookup?journal=Med+Educ+Online&title=Medical+student+debt+and+major+life+choices+other+than+specialty&author=J+Rohlfing&author=R+Navarro&author=OZ+Maniya&author=BD+Hughes&author=DK+Rogalsky&volume=19&publication_year=2014&pages=25603&pmid=25391976&)]

29. Sterbling HM, Molena D, Rao SR, Stein SL, Litle VR. Initial report on young cardiothoracic surgeons' first job: from searching to securing and the gaps in between. *J Thorac Cardiovasc Surg.*2019;**158**(2):632–41 e3. [[PMC free article](https://www.ncbi.nlm.nih.gov/pmc/articles/PMC8142151/)] [[PubMed](https://pubmed.ncbi.nlm.nih.gov/30857819)] [[Google Scholar](https://scholar.google.com/scholar_lookup?journal=J+Thorac+Cardiovasc+Surg.&title=Initial+report+on+young+cardiothoracic+surgeons%27+first+job:+from+searching+to+securing+and+the+gaps+in+between&author=HM+Sterbling&author=D+Molena&author=SR+Rao&author=SL+Stein&author=VR+Litle&volume=158&issue=2&publication_year=2019&pages=632-41+e3&pmid=30857819&)]

30. Sanfey H, Crandall M, Shaughnessy E, Stein SL, Cochran A, Parangi S, et al. Strategies for identifying and closing the gender salary gap in surgery. *J Am Coll Surg.*2017;**225**(2):333–338. [[PubMed](https://pubmed.ncbi.nlm.nih.gov/28400299)] [[Google Scholar](https://scholar.google.com/scholar_lookup?journal=J+Am+Coll+Surg&title=Strategies+for+identifying+and+closing+the+gender+salary+gap+in+surgery&author=H+Sanfey&author=M+Crandall&author=E+Shaughnessy&author=SL+Stein&author=A+Cochran&volume=225&issue=2&publication_year=2017&pages=333-338&pmid=28400299&)]

31. Cantor JC, Baker LC, Hughes RG. Preparedness for practice. young physicians' views of their professional education. *JAMA.*1993;**270**(9):1035–1040. [[PubMed](https://pubmed.ncbi.nlm.nih.gov/8350444)] [[Google Scholar](https://scholar.google.com/scholar_lookup?journal=JAMA&title=Preparedness+for+practice.+young+physicians%27+views+of+their+professional+education&author=JC+Cantor&author=LC+Baker&author=RG+Hughes&volume=270&issue=9&publication_year=1993&pages=1035-1040&pmid=8350444&)]

32. Holak EJ, Kaslow O, Pagel PS. Facilitating the transition to practice: a weekend retreat curriculum for business-of-medicine education of United States anesthesiology residents. *J Anesth.*2010;**24**(5):807–810. [[PubMed](https://pubmed.ncbi.nlm.nih.gov/20563736)] [[Google Scholar](https://scholar.google.com/scholar_lookup?journal=J+Anesth&title=Facilitating+the+transition+to+practice:+a+weekend+retreat+curriculum+for+business-of-medicine+education+of+United+States+anesthesiology+residents&author=EJ+Holak&author=O+Kaslow&author=PS+Pagel&volume=24&issue=5&publication_year=2010&pages=807-810&pmid=20563736&)]

33. Williams LL. Teaching residents practice-management knowledge and skills: an in vivo experience. *Acad Psychiatry.*2009;**33**(2):135–138. [[PubMed](https://pubmed.ncbi.nlm.nih.gov/19398627)] [[Google Scholar](https://scholar.google.com/scholar_lookup?journal=Acad+Psychiatry&title=Teaching+residents+practice-management+knowledge+and+skills:+an+in+vivo+experience&author=LL+Williams&volume=33&issue=2&publication_year=2009&pages=135-138&pmid=19398627&)]

34. Adiga K, Buss M, Beasley BW. Perceived, actual, and desired knowledge regarding Medicare billing and reimbursement. A national needs assessment survey of internal medicine residents. *J Gen Intern Med.*2006;**21**(5):466–470. [[PMC free article](https://www.ncbi.nlm.nih.gov/pmc/articles/PMC1484800/)] [[PubMed](https://pubmed.ncbi.nlm.nih.gov/16704389)] [[Google Scholar](https://scholar.google.com/scholar_lookup?journal=J+Gen+Intern+Med&title=Perceived,+actual,+and+desired+knowledge+regarding+Medicare+billing+and+reimbursement.+A+national+needs+assessment+survey+of+internal+medicine+residents&author=K+Adiga&author=M+Buss&author=BW+Beasley&volume=21&issue=5&publication_year=2006&pages=466-470&pmid=16704389&)]

35. Stubbe DE. Preparation for practice: child and adolescent psychiatry graduates' assessment of training experiences. *J Am Acad Child Adolesc Psychiatry.*2002;**41**(2):131–139. [[PubMed](https://pubmed.ncbi.nlm.nih.gov/11837402)] [[Google Scholar](https://scholar.google.com/scholar_lookup?journal=J+Am+Acad+Child+Adolesc+Psychiatry&title=Preparation+for+practice:+child+and+adolescent+psychiatry+graduates%27+assessment+of+training+experiences&author=DE+Stubbe&volume=41&issue=2&publication_year=2002&pages=131-139&pmid=11837402&)]

36. Lusco VC, Martinez SA, Polk HC., Jr Program directors in surgery agree that residents should be formally trained in business and practice management. *Am J Surg.*2005;**189**(1):11–13. [[PubMed](https://pubmed.ncbi.nlm.nih.gov/15701483)] [[Google Scholar](https://scholar.google.com/scholar_lookup?journal=Am+J+Surg&title=Program+directors+in+surgery+agree+that+residents+should+be+formally+trained+in+business+and+practice+management&author=VC+Lusco&author=SA+Martinez&author=HC+Polk&volume=189&issue=1&publication_year=2005&pages=11-13&pmid=15701483&)]

37. Williford LE, Ling FW, Summitt RL, Jr, Stovall TG. Practice management in obstetrics and gynecology residency curriculum. *Obstet Gynecol.*1999;**94**(3):476–479. [[PubMed](https://pubmed.ncbi.nlm.nih.gov/10472882)] [[Google Scholar](https://scholar.google.com/scholar_lookup?journal=Obstet+Gynecol&title=Practice+management+in+obstetrics+and+gynecology+residency+curriculum&author=LE+Williford&author=FW+Ling&author=RL+Summitt&author=TG+Stovall&volume=94&issue=3&publication_year=1999&pages=476-479&pmid=10472882&)]

38. Tsai JC, Lee PP, Chasteen S, Taylor RJ, Brennan MW, Schmidt GE. Resident physician mentoring program in ophthalmology: the tennessee experience. *Arch Ophthalmol.*2006;**124**(2):264–267. [[PubMed](https://pubmed.ncbi.nlm.nih.gov/16476897)] [[Google Scholar](https://scholar.google.com/scholar_lookup?journal=Arch+Ophthalmol&title=Resident+physician+mentoring+program+in+ophthalmology:+the+tennessee+experience&author=JC+Tsai&author=PP+Lee&author=S+Chasteen&author=RJ+Taylor&author=MW+Brennan&volume=124&issue=2&publication_year=2006&pages=264-267&pmid=16476897&)]

39. Gill JB, Schutt RC., Jr Practice management education in orthopaedic surgical residencies. *J Bone Joint Surg Am.*2007;**89**(1):216–219. [[PubMed](https://pubmed.ncbi.nlm.nih.gov/17200329)] [[Google Scholar](https://scholar.google.com/scholar_lookup?journal=J+Bone+Joint+Surg+Am&title=Practice+management+education+in+orthopaedic+surgical+residencies&author=JB+Gill&author=RC+Schutt&volume=89&issue=1&publication_year=2007&pages=216-219&pmid=17200329&)]

40. Wichman CL, Netzel PJ, Menaker R. Preparing psychiatric residents for the "real world": a practice management curriculum. *Acad Psychiatry.*2009;**33**(2):131–134. [[PubMed](https://pubmed.ncbi.nlm.nih.gov/19398626)] [[Google Scholar](https://scholar.google.com/scholar_lookup?journal=Acad+Psychiatry&title=Preparing+psychiatric+residents+for+the+%22real+world%22:+a+practice+management+curriculum&author=CL+Wichman&author=PJ+Netzel&author=R+Menaker&volume=33&issue=2&publication_year=2009&pages=131-134&pmid=19398626&)]

41. Babitch LA. Teaching practice management skills to pediatric residents. *Clin Pediatr (Phila)*2006;**45**(9):846–849. [[PubMed](https://pubmed.ncbi.nlm.nih.gov/17041173)] [[Google Scholar](https://scholar.google.com/scholar_lookup?journal=Clin+Pediatr+(Phila)&title=Teaching+practice+management+skills+to+pediatric+residents&author=LA+Babitch&volume=45&issue=9&publication_year=2006&pages=846-849&pmid=17041173&)]

42. Chan S. Management education during radiology residency: development of an educational practice. *Acad Radiol.*2004;**11**(11):1308–1317. [[PubMed](https://pubmed.ncbi.nlm.nih.gov/15561581)] [[Google Scholar](https://scholar.google.com/scholar_lookup?journal=Acad+Radiol&title=Management+education+during+radiology+residency:+development+of+an+educational+practice&author=S+Chan&volume=11&issue=11&publication_year=2004&pages=1308-1317&pmid=15561581&)]

43. David RA, Reich LM. The creation and evaluation of a systems-based practice/managed care curriculum in a primary care internal medicine residency program. *Mt Sinai J Med.*2005;**72**(5):296–299. [[PubMed](https://pubmed.ncbi.nlm.nih.gov/16184291)] [[Google Scholar](https://scholar.google.com/scholar_lookup?journal=Mt+Sinai+J+Med&title=The+creation+and+evaluation+of+a+systems-based+practice/managed+care+curriculum+in+a+primary+care+internal+medicine+residency+program&author=RA+David&author=LM+Reich&volume=72&issue=5&publication_year=2005&pages=296-299&pmid=16184291&)]

44. Crites GE, Schuster RJ. A preliminary report of an educational intervention in practice management. *BMC Med Educ.*2004;**4**:15. [[PMC free article](https://www.ncbi.nlm.nih.gov/pmc/articles/PMC520819/)] [[PubMed](https://pubmed.ncbi.nlm.nih.gov/15380023)] [[Google Scholar](https://scholar.google.com/scholar_lookup?journal=BMC+Med+Educ&title=A+preliminary+report+of+an+educational+intervention+in+practice+management&author=GE+Crites&author=RJ+Schuster&volume=4&publication_year=2004&pages=15&pmid=15380023&)]

45. Bayard M, Peeples CR, Holt J, David DJ. An interactive approach to teaching practice management to family practice residents. *Fam Med.*2003;**35**(9):622–624. [[PubMed](https://pubmed.ncbi.nlm.nih.gov/14523656)] [[Google Scholar](https://scholar.google.com/scholar_lookup?journal=Fam+Med&title=An+interactive+approach+to+teaching+practice+management+to+family+practice+residents&author=M+Bayard&author=CR+Peeples&author=J+Holt&author=DJ+David&volume=35&issue=9&publication_year=2003&pages=622-624&pmid=14523656&)]

46. Yardley S, Westerman M, Bartlett M, Walton JM, Smith J, Peile E. The do's, don't and don't knows of supporting transition to more independent practice. *Perspect Med Educ.*2018;**7**(1):8–22. [[PMC free article](https://www.ncbi.nlm.nih.gov/pmc/articles/PMC5807269/)] [[PubMed](https://pubmed.ncbi.nlm.nih.gov/29383578)] [[Google Scholar](https://scholar.google.com/scholar_lookup?journal=Perspect+Med+Educ&title=The+do%27s,+don%27t+and+don%27t+knows+of+supporting+transition+to+more+independent+practice&author=S+Yardley&author=M+Westerman&author=M+Bartlett&author=JM+Walton&author=J+Smith&volume=7&issue=1&publication_year=2018&pages=8-22&)]

47. Higgins R, Gallen D, Whiteman S. Meeting the non-clinical education and training needs of new consultants. *Postgrad Med J.*2005;**81**(958):519–523. [[PMC free article](https://www.ncbi.nlm.nih.gov/pmc/articles/PMC1743321/)] [[PubMed](https://pubmed.ncbi.nlm.nih.gov/16085744)] [[Google Scholar](https://scholar.google.com/scholar_lookup?journal=Postgrad+Med+J&title=Meeting+the+non-clinical+education+and+training+needs+of+new+consultants&author=R+Higgins&author=D+Gallen&author=S+Whiteman&volume=81&issue=958&publication_year=2005&pages=519-523&pmid=16085744&)]

48. Sachdeva AK, Flynn TC, Brigham TP, Dacey RG, Jr, Napolitano LM, Bass BL, et al. Interventions to address challenges associated with the transition from residency training to independent surgical practice. *Surgery.*2014;**155**(5):867–882. [[PubMed](https://pubmed.ncbi.nlm.nih.gov/24656857)] [[Google Scholar](https://scholar.google.com/scholar_lookup?journal=Surgery&title=Interventions+to+address+challenges+associated+with+the+transition+from+residency+training+to+independent+surgical+practice&author=AK+Sachdeva&author=TC+Flynn&author=TP+Brigham&author=RG+Dacey&author=LM+Napolitano&volume=155&issue=5&publication_year=2014&pages=867-882&pmid=24656857&)]

49. Griffin A, Abouharb T, Etherington C, Bandura T. Transitional to independent practice: a national enquiry into the educational support for newly qualified GPs. *Educ Primary Care.*2010;**21**:299–307. [[PubMed](https://pubmed.ncbi.nlm.nih.gov/20868545)] [[Google Scholar](https://scholar.google.com/scholar_lookup?journal=Educ+Primary+Care&title=Transitional+to+independent+practice:+a+national+enquiry+into+the+educational+support+for+newly+qualified+GPs&author=A+Griffin&author=T+Abouharb&author=C+Etherington&author=T+Bandura&volume=21&publication_year=2010&pages=299-307&pmid=20868545&)]

50. Ecomopoulos K, Sun R, Garvey E, Ba’zzarelli A. Coaching and mentoring modern surgeons. *Bull Am Coll Surg.*2014;**99**:30–35. [[PubMed](https://pubmed.ncbi.nlm.nih.gov/25145045)] [[Google Scholar](https://scholar.google.com/scholar_lookup?journal=Bull+Am+Coll+Surg&title=Coaching+and+mentoring+modern+surgeons&author=K+Ecomopoulos&author=R+Sun&author=E+Garvey&author=A+Ba%E2%80%99zzarelli&volume=99&publication_year=2014&pages=30-35&)]

51. Straus SE, Chatur F, Taylor M. Issues in the mentor-mentee relationship in academic medicine: a qualitative study. *Acad Med.*2009;**2009**(84):135–139. [[PubMed](https://pubmed.ncbi.nlm.nih.gov/19116493)] [[Google Scholar](https://scholar.google.com/scholar_lookup?journal=Acad+Med&title=Issues+in+the+mentor-mentee+relationship+in+academic+medicine:+a+qualitative+study&author=SE+Straus&author=F+Chatur&author=M+Taylor&volume=2009&issue=84&publication_year=2009&pages=135-139&)]

52. Fang D, Moy E, Colburn L, Hurley J. Racial and ethnic disparities in faculty promotion in academic medicine. *JAMA.*2000;**284**:1085–1092. [[PubMed](https://pubmed.ncbi.nlm.nih.gov/10974686)] [[Google Scholar](https://scholar.google.com/scholar_lookup?journal=JAMA&title=Racial+and+ethnic+disparities+in+faculty+promotion+in+academic+medicine&author=D+Fang&author=E+Moy&author=L+Colburn&author=J+Hurley&volume=284&publication_year=2000&pages=1085-1092&pmid=10974686&)]

53. MacMillan TE, Rawal S, Cram P, Liu J. A journal club for peer mentorship: helping to navigate the transition to independent practice. *Perspectives on Medical Education.*2016;**5**(5):312–315. [[PMC free article](https://www.ncbi.nlm.nih.gov/pmc/articles/PMC5035278/)] [[PubMed](https://pubmed.ncbi.nlm.nih.gov/27631332)] [[Google Scholar](https://scholar.google.com/scholar_lookup?journal=Perspectives+on+Medical+Education&title=A+journal+club+for+peer+mentorship:+helping+to+navigate+the+transition+to+independent+practice&author=TE+MacMillan&author=S+Rawal&author=P+Cram&author=J+Liu&volume=5&issue=5&publication_year=2016&pages=312-315&pmid=27631332&)]

54. Kleinschmidt P, Addington-White J, Feldstein DA, Abraham V, Baier L. Increasing senior resident readiness to practice in primary care. *J Gen Intern Med.*2018;**33**:S712–S713. [[Google Scholar](https://scholar.google.com/scholar_lookup?journal=J+Gen+Intern+Med&title=Increasing+senior+resident+readiness+to+practice+in+primary+care&author=P+Kleinschmidt&author=J+Addington-White&author=DA+Feldstein&author=V+Abraham&author=L+Baier&volume=33&publication_year=2018&pages=S712-S713&)]

55. Gephart MH, Schaffer R, Katznelson L, Piro N. Transition-to-Practice Curriculum in Graduate Medical Education. *J Neurosurgery.*2017;**126**(4):A1398–A. [[Google Scholar](https://scholar.google.com/scholar_lookup?journal=J+Neurosurgery&title=Transition-to-Practice+Curriculum+in+Graduate+Medical+Education&author=MH+Gephart&author=R+Schaffer&author=L+Katznelson&author=N+Piro&volume=126&issue=4&publication_year=2017&pages=A1398-A&)]
