# Supplementary material for: Transition to practice curriculum for general internal medicine physicians: scoping review and Canadian national survey
Source: BMC Med Educ. 2022 Aug 9;22:609. doi: 10.1186/s12909-022-03673-4 (PMC9361703; doi:10.1186/s12909-022-03673-4)
Supplement: Supplementary file 3 — Additional file 3. [file 12909_2022_3673_MOESM3_ESM.docx]

**SUPPLEMENTARY TABLE 2**

| **RESOURCE** | **DESCRIPTION** |
| --- | --- |
| CMPA* Module 1 | Included parts on: |
|  | (i) Understanding Harm: How harm occurs |
|  | (ii) Just culture: Safer patients - every patient, every time |
|  | (iii) Systems: Systems thinking for safe care |
|  | (v) Accountability: Being professionally responsible |
|  | (v) Quality and safety improvement: Making things better |
|  | (vi) Governance: Regulation in Healthcare |
|  | (vii) Legal liability: When harm from healthcare delivery occurs |
|  | (viii) Test yourself: Check your knowledge |
| CMPA Module 2 (Teams) | (i) Healthcare teams: Driven by the needs of the patient |
|  | (ii) Safe teamwork: Successful teams reduce risk |
|  | (ii) Delegation and supervision: Responsibilities of supervisors and trainees |
|  | (v) Medical-legal lessons: From the CMPA files |
|  | (v) Test yourself: Check your knowledge |
| CMPA Module 3 (Communication) | (i) Patient-centred communication: Fostering understanding |
|  | (ii) Privacy and confidentiality: Protecting patient information |
|  | (iii) Informed Consent: more than a signature |
|  | (iv) Informed Discharge: Alerting patients to warning symptoms and signs |
|  | (v) Team communication: Let's talk |
|  | (vi) Handovers: Transferring care to others |
|  | (vii) Consultations and referals: Improving the referral-consultation process |
|  | (viii) Documentation: Document, document, document! |
|  | (ix) Test yourself: Check your knowledge |
| CMPA Module 4 (Managing Risk) | (i) The diagnostic process: a complex task |
|  | (ii) Diagnostic tips: Reducing medical-legal difficulties |
|  | (iii) Reducing risk in surgery: Patient safety during operative treatment |
|  | (iv) Medication risks: Safe care and medications |
|  | (v) Additional tips: Further insights |
|  | (vi) Test yourself: Check your knowledge |
| CMPA Module 5 (Human Factors) | (i) Defining human factors: Human characteristics and workplaces |
|  | (ii) Challenge to diagnosing: Patient factors, physician factors and system factors |
|  | (iii) Cognitive biases: Influences on decision making |
|  | (iv) Situational awareness: What is going on around you? |
|  | (v) Equipment and technology: Using equipment and technology safely |
|  | (vi) Other workplace factors: Influences on patient care |
|  | (vii) Human factors engineering: Designing an safe care environment |
|  | (viii) Test yourself: Check your knowledge |
| CMPA Module 6 (Adverse Events) | (i) Errors and matters of judgment |
|  | (ii) Disclosure |
|  | (iii) Quality improvement |
|  | (iv) Managing stress |
|  | (v) Test yourself: Check your knowledge |
| CMPA Module 7 (Professionalism) | (i) Values, commitment and expectations |
|  | (ii) Being honest: Practicing with integrity and trustworthiness |
|  | (iii) Cultural safety: Respect for patients and families |
|  | (iv) Behavior: Courtesy and respect |
|  | (v) Dealing with conflict: Working together |
|  | (vi) Respecting boundaries: Staying on side |
|  | (vii) Social media: Sharing responsibly |
|  | (viii) Test yourself: Check your knowledge |
| CMPA tips on social media use | https://www.cmpa-acpm.ca/en/advice-publications/browse-articles/2014/top-10-tips-for-using-social-media-in-professional-practice |
| Humanitarian Leadership Academy | https://www.humanitarianleadershipacademy.org/ |
|  | Relevant free courses available on conflict resolution, change management, wellness and resilience for frontline workers and manager, managing yourself and your time, managing your priorities |
| Negotiating physician salaries | https://hbr.org/2014/04/15-rules-for-negotiating-a-job-offer AND |
|  | https://www.kevinmd.com/blog/2019/02/physician-negotiating-go-get-what-youre-worth.html |
| Learning financial and administrative aspects of practice management | PAMS (Practice Advisory and Management Services) through the OMA |
|  | Health Force Ontario also has a branch: |
|  | http://www.healthforceontario.ca/UserFiles/file/PracticeOntario/TiPS/TiPS-BSM-EN.pdf |
| Creating learning plan for continuous professional development | Each provincial licensing authority has regulations regarding needs to maintain certification, as CPD |
|  | https://www.cpso.on.ca/admin/CPSO/media/Documents/physician/your-practice/quality-in-practice/continuing-professional-development/cpd-faq.pdf |
| Locations (in Canada) to find physician jobs | https://csim.ca/careers/ |
|  | https://ca.indeed.com |
|  | https://www.workopolis.com/ |
|  | https://www.drcareers.ca/jobs/ |
|  | https://www.glassdoor.ca/Job/ |
